# Transition to an in-facility electronic Tuberculosis register: Lessons from a South African pilot project

**DOI:** 10.4102/sajhivmed.v21i1.1025

**Published:** 2020-01-16

**Authors:** Hanlie Myburgh, Remco P.H. Peters, Theunis Hurter, Cornelius J. Grobbelaar, Graeme Hoddinott

**Affiliations:** 1 Desmond Tutu TB Centre, Department of Paediatrics and Child Health, Faculty of Medicine and Health Sciences, Stellenbosch University, Cape Town, South Africa; 2 Anova Health Institute, Johannesburg, South Africa

**Keywords:** TB programme, systems integration, monitoring and evaluation, roles and responsibilities, HIV

## Abstract

**Background:**

South Africa has one of the highest incidences of Tuberculosis (TB) globally. High co-morbid HIV prevalence complicates TB management and treatment outcomes. Growing evidence suggests that integrating the TB and HIV programmes will improve the overall results.

**Objectives:**

To describe how TB programme staff at various levels of the South African health system responded to the transition from a paper-based to an electronic register of TB data integrated with HIV programme data.

**Method:**

Three primary health service facilities in the Cape Winelands district, Western Cape province, South Africa served as pilot sites for implementation. Semi-structured interviews were conducted with 21 TB programme staff purposively selected at facility, sub-district, district and provincial levels of the health system, based on their involvement in implementing electronic TB data. An objective-driven thematic frame was used to analyse the data.

**Results:**

Fears about the transition included reductions in data quality, changes to the status quo and a lack of computer literacy. Participants acknowledged benefits of reduced workloads, speed of accessing patient-level data and click-of-a-button reporting. Three factors influenced the ease of adopting the new system: firstly, implementation challenged the vertical position of the TB programme, TB data and staff’s conventional roles and responsibilities; secondly, perceptions of the paper-based register as functional and reliable made the transition to electronic seem unnecessary; and thirdly, lack of a process of change management challenged staff’s ability to internalise the proposed change.

**Conclusion:**

A process of change management is critical to facilitate the efficiency and effectiveness with which the electronic in-facility TB register is implemented.

## Introduction

South Africa has one of the highest burdens of Tuberculosis (TB) globally.^1,2^ In 2017, the estimated incidence of drug-susceptible TB (DS-TB) was 567 per 100 000 persons, and there were approximately 78 000 deaths from TB-related causes.^3,4^ The close relationship between TB and HIV (> 60% of TB patients are also living with HIV) further complicates TB management and treatment outcomes.^2,5,6^ Growing evidence suggests that integrating the TB and HIV programmes will improve overall outcomes and reduce mortality.^1,6,7,8^

## Background

**FIGURE 1:**
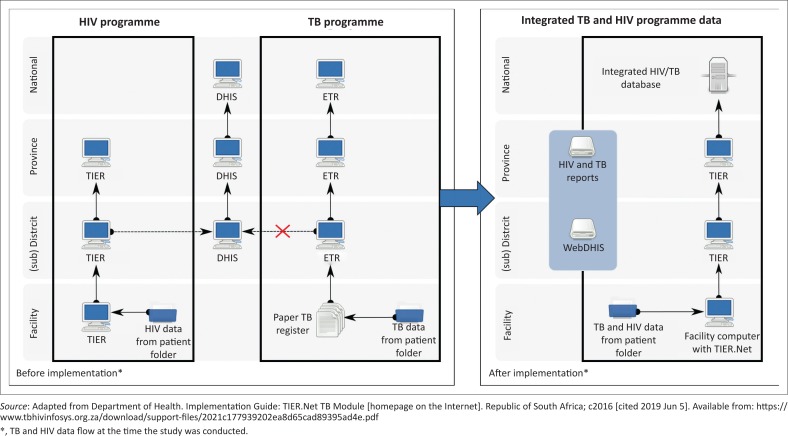
The data flow of TB and HIV programme data before and after the implementation of the TB module in TIER.Net.^12^ Prior to implementation, the TB and HIV programme comprised two separate systems (TIER, ETR), each maintained on separate hardware with its own support structure; co-infected patients were tracked separately. The TB system emphasises data reporting with the use of paper registers, and facility level staff depend on sub-district TB coordinators for programme feedback; the HIV system (TIER.Net) combines immediate, real-time access to individual and aggregated HIV data for patient management and programme reporting, and is integrated with the DHIS. After implementation, TB and HIV programme data flow up through TIER.Net and are consolidated into one database. TB and HIV programme data are available at all levels of the health system for querying and reporting (national through to facilities) and is integrated with the WebDHIS system.

In South Africa, TB and HIV programmes and health information systems are implemented as vertical and siloed systems and have largely retained this separation.^1,2,5^ Since 1995, the National TB Programme has been supported by a central standardised recording system to monitor TB case rates and treatment outcomes. This system comprises paper-based registers at facility level. An Electronic TB Register (ETR.Net) for DS-TB at sub-district, district, provincial and national levels was added in 2005.^1,9,10,11^ In 2014, the National Department of Health of South Africa took a decision to integrate the TB and HIV information systems at facility level into a single non-networked electronic system called TIER.Net.^12^ Since 2010, TIER.Net has been serving as the primary monitoring platform for the national antiretroviral treatment (ART) programme^13^ and was incrementally expanded to include HIV testing and pre-ART data modules. TIER.Net is used to capture patient-level HIV information at facility level and is integrated with the district health information system (DHIS) for reporting various programme data from sub-district to national levels. In contrast, TB programme data remained separate from other health programmes, where TB nurses capture patient information into facility level paper-based TB registers (Figure 1). Pages from the paper-based registers are sent to the sub-district administrative level where they are captured into ETR.Net. TB coordinators validate the captured data and refer queries back to the facilities. They also provide quarterly feedback to facilities and TB managers, and submit a dispatch of the data to the district level, from where it is sent to province, and finally to national level to generate annual reports. With the integration of TB and HIV programme data, a specially developed TB module for TIER.Net would supplant the paper-based TB register at facility level. In theory, this would allow TB programme staff at facility level immediate and easy access to individual and aggregated TB data. The introduction of the TB module is also the first step in decentralising TB programme data. Specifically, introduction of the TB module would shift the programme from one that performs surveillance only to one that uses real-time data for patient management and is integrated with the DHIS used for overall health programme reporting.

In this qualitative study, we retrospectively describe how TB programme staff working at various levels of the South African health system responded to the transition from a paper-based to an electronic TB data system at facility level prior to its widespread adoption and implementation. We consider the need to prioritise change management in health services implementation and the unique challenges posed by the history of the TB programme for data and service integration.

## Methods

### Study design and setting

The Department of Health identified three primary health service facilities in the Cape Winelands district in the Western Cape Province, South Africa, to serve as pilot sites for implementing electronic TB data at facility level. The sites were each located in different sub-districts and differed with respect to TB caseload, TB staff component and programmatic services offered. This qualitative evaluation was conducted independently from the implementation process.

### Sampling and data collection

Data were collected between July and September 2016, one year after implementation had started. Participants were purposively selected as key informants at facility, sub-district, district and provincial levels of the provincial health system based on their involvement in the implementation process of the TB module in TIER.Net in a decision-making, managerial and/or implementation capacity. Participants included:

- managers at facility, sub-district, district and provincial levels of the health system (*n* = 12) who were involved in the TB programme and health information in a managerial and decision-making capacity
- administrative staff at facility level (*n* = 2) who were responsible for electronically capturing health information for various health programmes, including the TB programme
- clinical TB staff or nurses at facility level (*n* = 6) who had experience using the paper TB registers and implementing the TB module in TIER.Net in each facility, including capturing TB data into the electronic register
- an implementing partner from the Anova Health Institute (*n* = 1) who provided extensive support to facility and sub-district level staff during implementation in all three pilot sites.

To maintain their anonymity all manager-participants are referred to as TB managers in the results regardless of their position in the health system. Discussions were conducted in participants’ preferred language by two bilingual (Afrikaans and English) researchers using a semi-structured discussion guide. Interviews were audio recorded and ranged from 20 min to 90 min. Interview questions were about participants’ recollections of their experiences of TB programme data with the paper-based system, the transition to an electronic system and their current experiences with the electronic system. The evaluation of the pilot project was funded by the Anova Health Institute, which was the implementing partner at the time of the study. To mediate potential desirability bias in participants’ responses during interviews, the researchers conducting the interviews were external to the organisation.

### Data analysis

Audio recordings were summarised and transcribed by the researchers. An objective-driven thematic frame was used to explore the data – namely aspects of the health information system that *could be influenced by* transition from a paper-based to an electronic TB patient register (e.g. resources, data flows, decision-making and accountability)^14,15^, as well as contextual health systems factors that *could influence* transition to the electronic TB register. Key ideas from the data were grouped into:

- contextual factors
- process-related changes during the transition
- recommendations for facilitating efficiency and effectiveness.

These findings were discussed amongst the authors, who drew on their experiences of implementing the TB programme and health information systems to interpret the data.

### Ethical onsiderations

Ethical clearance for this study was obtained from the University of Stellenbosch’s Health Research Ethics Committee, and an informed consent process was followed with each participant (Ethical Clearance No. N16/02/024).

## Results

Participants shared conflicting feelings about the transition to an electronic in-facility TB register, describing not only their anxieties around the transition but also acknowledging its benefits. Specifically, participants expressed fears over reduction in data quality, uncertainty over changes to the status quo and, for some facility level staff, insecurity regarding their ability to use an unfamiliar and electronic system. Participants referred to such challenges while describing positive experiences, such as significantly reduced workloads, speed of accessing patient-level data and click-of-a-button reporting. Our results report on three key contextual factors emerging from our interviews that gave rise to the conflicting sentiments that influenced the ease of adopting the facility level electronic TB register: (1) the position of TB programme and programme data, (2) perceptions about the new and old systems and (3) how acceptance of the new system was facilitated.

### Position of the tuberculosis programme and tuberculosis programme data at the facility

The historically siloed nature of the TB programme and data flow in South Africa enabled TB clinicians and managers careful control of programme data for surveillance purposes using paper-based registers. TB programme staff positioned themselves as ‘TB champions’, that is, as custodians of TB data, which they entered, tallied and then appropriated, and this led to them having a vested interest in the status quo of ‘their’ paper-based system. The transition to an electronic in-facility register signalled a shift in how the TB programme would be controlled, allowing more involvement of facility level staff in data entry and maintenance, and signalling a loss of control as the data would be available to a much broader audience. Excerpts from interviews illustrate the shift in power with introduction of the register:

‘There is one person in the clinic who completes the [*paper*] register; there is one person in the clinic who understands TB data. And all of a sudden [*with introduction of the electronic TB register*], the clerk must become involved, and more than one clerk, and more than one staff member.’ (Participant 19, female, TB manager, 13 September 2016)

‘In the olden days you felt like those old Sisters lording over everything – they can ask you anything, you know everything, you understand everything. And [*with the electronic TB register*] I don’t know it.’ (Participant 7, female, nurse, 09 September 2016)

The electronic register would allow facilities to query and clean their own data before submission in upward data flow, with sub-district TB coordinators taking on a less hands-on oversight role than they had before. This role change and the perceived effects of the transition on data quality raised anxieties:

‘Eighty percent of [*TB coordinators*’] work was ETR, was TB data. Data, data analysis, and data validation. Now we come and say that there is a possibility that we’ll take the ETR away because we want to do better patient management. That’s the anxiety – what about us now? What is our role? They don’t understand, they’ll still have a role in data. The role just needs to be clarified.’ (Participant 20, male, TB manager, 15 September 2016)

‘I kicked against the [*electronic*] system…because I felt that I was … a safety net [*for data quality*].’ (Participant 11, female, TB manager, 05 August 2016)

In one instance, fears over reduction in data quality caused a manager to undermine the register’s implementation:

‘[*The TB manager*] didn’t want us to spend time on the computer [*and implement the register*], just wanted the paper. We decided that we’re going to continue [*to implement*], we’re going to show them it works. Show them how we print reports, how quick it is.’ (Participant 1, female, clerk, 01 July 2016)

To allay fears over TB case registration and maintenance of data quality, all three facilities kept parallel paper-based and electronic registers at the start of implementation and monthly data audits were conducted throughout. While some TB managers continued to hold apprehensions over data quality, they also saw the potential of the electronic register to increase ownership of TB data at facility level:

‘I hope and trust that ownership [*of the data*] will be better because the data is not going far away to someone who captures it, the data is here in my clinic and I capture it myself.’ (Participant 13, male, TB manager, 25 August 2016)

Prior to the transition to the electronic register, using TB data for patient management required manual interrogation of patient folders to identify patients who missed sputum collection or who were experiencing treatment interruptions. This was labour and time-intensive and could not be regularly conducted by the three facilities without support. Yet, the notion that the electronic TB register offered click-of-a-button in-facility access to data for patient management and improved reporting was not realised at initial introduction:

‘[*The implementing partner*] told us everything that we see now: ”You will easily see that patients are late.“ We said ”We won’t, we’ll still have to go through the folders.” Everything he said is [*true/we were wrong*].’ (Participant 6, female, nurse, 19 August 2016)

### Perceptions about the ‘new’ electronic and ‘old’ paper-based register

Participants across all levels of the health system expressed familiarity with and confidence in the ETR.Net surveillance system and the paper-based registers that support it:

‘You can go to anyone in the Department of Health in the Western Cape and they will tell you that the only reliable data is TB data. With all the mistakes in the systems [*of other health programmes*] the only reliable data is TB data.’ (Participant 19, female, TB manager, 13 September 2016)

‘It was difficult for [*the nurses*] to let go of those papers. They were clinging to their register, ”Don’t take my register away!“’ (Participant 9, female, TB manager, 23 August 2016)

Accordingly, when the electronic in-facility TB register was introduced, some participants felt that it was an unnecessary change as it replaced a working system. Despite their apprehensions, managers in the TB programme recognised the transition to an electronic in-facility register as a logical progression in management of TB data. This related to the integration of TB programme data with general health information management, and to the broader notion that the TB programme should move with the time:

‘Anyone looking for TB data in the country must get data from the TB programme (and not from Health Information Management like with all other programmes). Integrated systems is the answer.’ (Participant 19, female, TB manager, 13 September 2016)

‘All other [*programmes*] are on [*electronic*] systems. That’s why TB must move away from paper-based. It might get resistance from some of the clinics, but usually it’s because people don’t understand.’ (Participant 13, male, TB manager, 25 August 2016)

Some participants expressed serious concerns about the integration of TB data with the existing HIV data infrastructure, TIER.Net. For some, their siloed work had given them little to no experience with the TIER.Net software, while others’ concerns were informed by the gaps they perceived in TIER.Net’s HIV and ART modules, which negatively influenced confidence in the new system’s ability to effectively maintain TB data:

‘We have a lot of work to do on the quality of capturing [*HIV programme data*] on TIER.Net, now we add the additional burden of TB … How can you go from point A to point B if your point A things aren’t correct yet?’ (Participant 11, female, TB manager, 05 August 2016)

This first pilot implementation of the electronic in-facility TB register highlighted some flaws in the software, producing erroneous reports on key TB indicators. This caused some participants to question the integrity of the new programme, and TB coordinators felt that they were responsible for resolving technical issues despite first and foremost being clinicians. Despite these challenges, regular meetings of TB programme stakeholders during implementation and training of in-facility staff (clerks and clinicians) on the electronic TB register kept momentum for implementation.

### How acceptance of the ‘new’ system was facilitated

In preparing facilities as implementation sites, efforts focussed largely on coalface implementers. Managers were primarily involved to follow due consultative process rather than as advisors and decision-makers in the implementation process. Department of Health implementers and implementing partners supported the transition by training clerks and TB clinicians on the electronic register and completion of clinical stationery, resource allocation (computers and additional staff during back-capturing active TB clients) and continuous feedback meetings during implementation. While some participants felt that these processes were sufficient, others expressed anxieties about how the decision to pilot the electronic facility level TB register was taken, discussions on how this change would be implemented and the broader implications for the TB programme and their roles:

‘It’s a paradigm shift, how we used to work in the past, and now we don’t work like that anymore. It’s tough, because many colleagues don’t trust the process; they’re used to a different process. It is our responsibility as senior managers to turn those heads.’ (Participant 20, male, TB manager, 15 September 2016)

Given the breadth of the proposed change that some participants felt the electronic facility level TB register ushered in, participants at sub-district and district management levels spoke about the need for change management:

‘When introducing something, come with change management to enable the people to grasp it and to internalise it. You get it today, and tomorrow must implement it. And that’s why people put up these walls. Resistance, resistance.’ (Participant 14, female, TB manager, 05 August 2016)

For staff at facility level, one of the most challenging aspects of implementation was related to their historic use of paper-based registers and subsequent underexposure and distrust of technology. Some participants also voiced concerns over the safety of electronic data during power outages, in case of computer theft and possible system failures. As such, computer literacy at facility level was a consistent concern of participants at all levels of the health system:

‘I think one of the shortcomings [*in rolling out further*] will be that colleagues aren’t excited about technology or that they are not ready to embrace computers.’ (Participant 12, male, TB manager, 23 August 2016)

The question ‘Who is best-placed to capture TB data?’ divided TB stakeholders into two camps: those advocating for TB clinicians to continue to capture data and those advocating for the responsibility to be handed over to clerks as is the practice in the HIV programme. In the two facilities with smaller patient numbers, the TB clinicians had quickly become adept at capturing TB data into the electronic register and drawing reports. At the time of the evaluation, clinicians in all three facilities were either responsible for or assisting with capturing TB data and were regularly accessing reports on the system.

## Discussion

There are numerous factors that influence transition from paper to electronic records and information systems in health services.^13,16,17^ These include organisational culture, for instance, readiness of the organisation and its end users to adopt a new technology,^18,19^ the ability of the innovation to integrate with existing, conventional workflows or to require changes to it,^19,20,21^ and more practical aspects such as computer literacy of staff which may influence how confident they feel to successfully implement the innovation.^15^ As such, even a seemingly simple replacement of a paper-based register with an electronic one may bring about important shifts in power for different users by requiring adapted skillsets, resulting in complex changes to the status quo. These factors ultimately shape the response of those affected by the proposed change.^22^

While the introduction and potential of electronic TB data at facility level can be considered an important step forward for the TB programme, many of the participants recounted strong initial reactions and resistance towards the proposed change that affected the efficiency and effectiveness of implementation. These anxieties and resistances were concerned with: firstly, the vertical position of the TB programme and TB programme data which ushered in changes to staff’s conventional, familiar roles and responsibilities; secondly, with perceptions about the ‘new’ electronic register as deleterious and unnecessary and the ‘old’ paper-based register as functional and reliable; and thirdly how adoption of the new register would be facilitated, which participants felt lacked a process whereby they could internalise the proposed change.

We make three recommendations for facilitating the transition to an electronic TB register at facility level in South Africa and for moving to integrated electronic systems in general.

Firstly, implementers must invest in a process of change management alongside the transition to electronic facility level TB data in South Africa. Kuhn and Giuse define change management as ‘the process of assisting individuals and organizations in passing from an old way of doing things to a new way of doing things’.^23^ To be successful, a change management process should involve the management of the practical aspects of the change (e.g. resources and training), and should address how the change might challenge the sense of security, confidence and identity that individuals associate with the conventional or old way of doing things.^24,25^ In the South African example, such a change management process must endeavour to achieve buy-in across all levels of the healthcare system by identifying the individuals or groups who will be affected by the change, and creating a space in which their anxieties can be voiced, acknowledged and addressed. This process could be facilitated by showcasing experiences and outcomes from pilot sites and providing practical examples of how challenges can be mediated and resolved. In other TB treatment contexts, such a change management process should involve prior formative research, which can include desk research, to establish the health and political context within which the TB programme is required to transition. In particular, this involves establishing how existing TB programmes and systems function, the relational nuances between people and programmes that might affect implementation (as is the case between the HIV and TB programmes in South Africa) and people’s loyalties to particular ways of operating within the TB programme.

Secondly, individuals driving implementation should include individuals from within the TB programme in order to bring expert knowledge of the existing system and to lend credibility to the proposed change. Implementers should directly address the potential challenges of transitioning, work with staff to set realistic expectations of individuals’ roles and responsibilities and how these may change with implementation, and ensure that they are communicated effectively. At each facility, staff should be allowed to tailor some elements of implementation to their local contexts, for instance, the decision about who is best placed to capture TB data, and provide support to develop sustainable plans for maintaining the data. The following aspects of implementation should be addressed: the rationale for the transition, in particular, the limitations of maintaining separate programme data and the possibilities opened up for improving TB patient management and programme outcomes with decentralisation and integration of TB data with those of other health programmes; anticipate implementers’ potential distrust and discomfort with the introduction of the electronic TB register, potential fears about losing data quality, and their familiarity with and trust in the functionality of the paper-based register and the ETR.Net system. Also pre-emption and discussion of the process of identifying and reporting flaws and compatibility issues in the software, and detailing of the support that is available if such issues were to arise.

Thirdly, it must be recognised that data use and analysis by facility managers and TB nurses will take time to cultivate; it is necessary to understand that the electronic register frees up the hands of sub-district level TB coordinators to provide health systems strengthening support to facilities by, for instance, using data in real-time to check progress against targets.

Through highlighting key issues to address during implementation, our study contributes to informing wide-scale implementation of electronic TB data in South African health facilities, and can inform the implementation of electronic health information systems in favour of paper-based systems globally. There are two limitations to the study. Firstly, the study uses interviews with participants a year after the pilot project began to report on implementation, thus asking participants to recall their experiences rather than documenting their experiences in real-time. Secondly, this study was also limited by its focus on pilot sites in one health district in the Western Cape Province which is not necessarily representative of other settings within and outside of South Africa.

## Conclusion

South Africa is one of the first countries to pilot electronic TB data at facility level for programme monitoring. In order to facilitate the efficiency and effectiveness with which the register is implemented, it is critical that a process of change management occur alongside its continued rollout. This process must address the shift from a vertical to integrated health information system for the TB programme on one level, and on another, its particular integration with TIER.Net, the health information system used for monitoring and evaluating the South African HIV programme. While our findings in this study are largely context-specific, there are significant similarities across TB programmes as vertical or siloed surveillance programmes that could extend our findings’ relevance beyond South Africa, particularly to contexts with comparatively high HIV and TB burdens.
